# Is that a real oocyst? Insectary establishment and identification of *Plasmodium falciparum* oocysts in midguts of Anopheles mosquitoes fed on infected human blood in Tororo, Uganda

**DOI:** 10.1186/s12936-019-2922-8

**Published:** 2019-08-27

**Authors:** Alex K. Musiime, Joseph Okoth, Melissa Conrad, Daniel Ayo, Ismail Onyige, John Rek, Joaniter I. Nankabirwa, Emmanuel Arinaitwe, Moses R. Kamya, Grant Dorsey, Geert-Jan van Gemert, Sarah G. Staedke, Chris Drakeley, Teun Bousema, Chiara Andolina

**Affiliations:** 1 Infectious Diseases Research Collaboration, Kampala, Uganda; 2 Department of Medicine, San Francisco General Hospital, University of California, San Francisco, USA; 3 Department of Medicine, Makerere University College of Health Sciences, Kampala, Uganda; 4 Faculty of Infectious and Tropical Diseases, London School of Hygiene and Tropical Medicine, London, UK; 5 Department of Medical Microbiology, Radboud University Nijmegen Medical Centre, Nijmegen, The Netherlands; 6 Department of Immunology and Infection, London School of Hygiene and Tropical Medicine, London, UK

**Keywords:** Malaria, Oocyst, Transmission, *Plasmodium falciparum*, Gametocyte

## Abstract

**Background:**

The human infectious reservoir for malaria consists of individuals capable of infecting mosquitoes. Oocyst prevalence and density are typical indicators of human infectivity to mosquitoes. However, identification of oocysts is challenging, particularly in areas of low malaria transmission intensity where few individuals may infect mosquitoes, and infected mosquitoes tend to have few oocysts. Here, features that differentiate oocysts from other oocyst-like in mosquito midguts are explained and illustrated. In addition, the establishment and maintenance of infrastructure to perform malaria transmission experiments is described. This work may support other initiatives to set up membrane feeding infrastructure and guide oocyst detection in low transmission settings.

**Methods:**

In 2014, an insectary was developed and equipped in Tororo district, Uganda. A colony of *Anopheles gambiae* s.s. mosquitoes (Kisumu strain) was initiated to support infectivity experiments from participants enrolled in a large cohort study. Venous blood drawn from participants who were naturally infected with malaria parasites was used for membrane feeding assays, using 60–80 mosquitoes per experiment. Approximately 9–10 days after feeding, mosquitoes were dissected, and midguts were stained in mercurochrome and examined by light microscopy for *Plasmodium falciparum* oocysts and similar structures. In supportive experiments, different staining procedures were compared using in vitro cultured parasites.

**Results:**

A stable colony of the Kisumu strain of *An. gambiae* s.s. was achieved, producing 5000–10,000 adult mosquitoes on a weekly basis. Challenges due to temperature fluctuations, mosquito pathogens and pests were successfully overcome. Oocysts were characterized by: presence of malaria pigment, clearly defined edge, round shape within the mosquito midgut or on the peripheral tissue and always attached to the epithelium. The main distinguishing feature between artifacts and mature oocysts was the presence of defined pigment within the oocysts.

**Conclusions:**

Oocysts may be mistaken for other structures in mosquito midguts. Distinguishing real oocysts from oocyst-like structures may be challenging for inexperienced microscopists due to overlapping features. The characteristics and guidelines outlined here support identification of oocysts and reliable detection at low oocyst densities. Practical advice on sustaining a healthy mosquito colony for feeding experiments is provided. Following the reported optimization, the established infrastructure in Tororo allows assessments of infectivity of naturally infected parasite carriers.

## Background

There has been a marked reduction in malaria transmission intensity in sub-Saharan Africa due to the intensification of malaria control interventions, including long-lasting insecticidal nets (LLINs) and indoor residual spraying (IRS) [1]. Access to malaria diagnosis also increased, supported by an increased supply of rapid diagnostic tests (RDTs) [2]. Despite this success, malaria continues to pose a public health burden in many countries worldwide and especially Africa [2]. In 2017, 435,000 deaths due to malaria occurred globally of which 93% occurred in the African region [2]. The global strategy is to reduce global malaria burden by 90% by 2030 and eliminate malaria in at least 35 countries actively pursuing malaria elimination [3]. In Uganda, mass distribution of LLINs was conducted in 2013 and studies later showed that 80% of the population were using an LLIN [4]. The combination of LLINs and IRS was followed by substantial reductions in malaria burden in the Tororo district [5, 6], as also observed in other Ugandan areas with high transmission intensity [7].

As has been reported elsewhere, rapid gains in malaria control do not necessarily lead to malaria elimination [8, 9], and a large reservoir of parasites may persist in the human population [10]. Asymptomatic infections may be important sources of onward transmission to mosquitoes [10, 11]. The transmission of *Plasmodium* to mosquitoes depends on the presence of mature female and male gametocytes in the peripheral blood. Once ingested by blood-feeding *Anopheles* mosquitoes, gametocytes activate to form gametes that fuse to form a zygote. The zygote in turn becomes motile and elongated (ookinete), invading the midgut wall of the mosquito where it develops into an oocyst. The oocyst grows over time and, upon rupture, releases sporozoites that travel via haemolymph to the mosquito’s salivary glands and render the mosquito infectious to humans. Whilst the density of gametocytes is strongly associated with the likelihood of mosquitoes becoming infected [12, 13], transmission efficiency may differ between populations [12, 14] and is influenced by factors such as human immune responses [15] and treatment [13]. Malaria transmission thus needs to be quantified directly by allowing mosquitoes to feed on the skin of malaria-infected individuals or on venous blood drawn from infected individuals in direct membrane feeding assays (DMFA) [16, 17]. Such mosquito-feeding assays in combination with assessments of parasite and gametocyte carriage are highly informative to understand the contribution of different populations to onward transmission and the duration of infectiousness following successful malaria control.

To this purpose, a longitudinal cohort study was initiated in Nagongera sub-county, Tororo district in 2017 to longitudinally evaluate the role symptomatic and asymptomatic carriers in maintaining transmission. These types of studies are considered crucial to inform malaria elimination initiatives and need to be conducted at different endemicities [18]. It is a considerable challenge to set up the required entomology infrastructure and optimize mosquito feeding procedures to obtain reliable results. Here, the inception of DMFA in Tororo, Uganda is presented along with the key challenges that were experienced and factors that were considered important in the successful establishment of membrane feeding facilities to assess the human infectious reservoir for malaria. The aim of this study was to explain the lessons learnt during the optimization process and illustrate features that differentiate oocysts from other structures seen under light microscope in mosquito midguts that may resemble oocysts. Together, the current work may support the establishment of mosquito feeding experiments in other endemic settings.

## Methods

### Study site and insectary

This study was conducted in Nagongera sub-county, located in Tororo district in Eastern Uganda. Nagongera is a rural setting with historically high transmission intensity. The *Plasmodium falciparum* annual entomological inoculation rate (*Pf*AEIR) was 562 bites per person per year in June 2001–May 2002 and *Pf*AEIR and 125 in October 2011–September 2012, following a combination of interventions including a “test and treat policy” using RDTs, intermittent preventive therapy in pregnancy (IPTp), introduction of artemisinin-based combination therapy (ACT) for the treatment of uncomplicated *P*. *falciparum* malaria and vector control [19–22]. In December 2013, Tororo district achieved near universal (98%) coverage of LLINs, giving one free bed net per two individuals in each household. In December 2014; integrated vector management started by introducing IRS in addition to LLINs [23]. These interventions were followed by a rapid reduction of malaria and mosquito densities in these areas [6, 24]; mean monthly malaria incidence decreased from 95 cases per 1000 in 2013 to 36 in 2015 [6].

The insectary used for this study is located at a local health facility in Nagongera and consists of two refurbished, sealed, shipping containers (14 m × 2.4 m and 12 m × 2.4 m) that are divided into five different working areas for larvae rearing (one room, equipped with heater but not air-conditioned), adult maintenance (two rooms, equipped with heater, air conditioner controlled by a thermostat and humidifier), and two rooms for carrying out experiments and dissections. The insectary and an *Anopheles gambiae* s.s. colony was established in 2014 with eggs acquired from the Kisumu insectary (CGHR-KEMRI) in Kenya. *Anopheles gambiae* were reared in a temperature-controlled insectary. Human blood provided by volunteers from the local community was used for colony maintenance as attempts with cow blood were unsatisfactory. Whilst it was possible to let mosquitoes feed on cow blood, egg production was higher when fresh human blood was used and this approach was thus preferred. Human donor blood was fed to mosquitoes using 1 ml Haemotek^®^ feeders following heat-inactivation [25] at 43 °C for 15 min to prevent possible *P*. *falciparum* infectivity in case of malaria-infected volunteers. Two to three days post-feeding, egg bowls were placed in cages. To increase hatching rates, collected eggs were hatched in trays of approximately 3 l capacity illuminated by light bulbs. To prevent egg desiccation due to evaporation, squeeze bottles were used daily to spray water around the edges of trays. Newly emerged larvae were fed with three drops of Liquifry (Liquifry No1, food for baby egg-laying fish, Interpet, UK) food until larval first instar (L1) developed to second larval instar (L2). Larvae were transferred to larger basins of approximately 10 l capacity containing mineral drinking water and three floating food sticks (King British, Cichlid floating food sticks-with immune health booster IHB, UK) were added. When all food particles disappeared, three more sticks were added to the basin.

Initially, tap water was used to raise larvae. However, high mortality occurred sporadically when tap water was used, plausibly due to unknown contaminants or chemicals in the water. To avoid these uncertainties, bottled mineral drinking water was used instead, resulting in more predictable larvae growth and fewer incidents of high mortality. Every morning, larvae basins were placed in direct sunlight for up to 4 h. The increase in water temperature (from 25 °C to up to 38 °C) accelerated larvae growth. Adult mosquitoes emerged after approximately 7 days. The temperature of the adult room was maintained at approximately 25–27 °C and ~ 75–85% relative humidity by use of local heaters and a humidifier (Condair 505, Switzerland) and monitored continuously with a probe (TinyTag Plus 2 TGP-4500, UK). Approximately 1000–1500 adults were held in each locally made cage (30 × 30 × 30 cm) or commercial BugDorm cage (BugDorm-1 Insect rearing cage, 30 × 30 × 30 cm, BugDorm Store, Taichung, Taiwan). Mosquitoes that were used for experiments, and thus fed on potentially infectious blood, were kept in paper cups placed inside BugDorm cages for safety reasons.

### Direct membrane feeding assay

In preparation of feeding experiments, female mosquitoes that were 4–7 days old (i.e. post-emergence) were selected by placing a hand close to the cage or using a plastic bottle containing hot water at 38 °C and selecting aggressive mosquitoes by mouth aspirator. Following visual inspection of the selected mosquitoes to ensure no male mosquitoes were included, female *Anopheles* were placed in labelled paper cups of approximately 300 ml capacity, covered with netting and starved for approximately 4 h. For other colonies, longer starvation times may be needed to ensure high feeding rates [26]. For each experiment, a total of 60–80 female were used for DMFA; these were divided over two cups (maximum of 40 mosquitoes/cup). Venous blood was drawn from selected study participants in heparinized tubes (Vacutainer^®^ Plastic Lithium Heparin Tube, 4 ml) and two feeders were immediately filled with 0.5 ml of blood each. Water-jacket glass feeders (mini-feeder, Coelen Glastechniek, The Netherlands) with Parafilm were attached to a circulating water bath set to 38 °C to achieve a temperature of 36.5–37 °C in feeders along the feeder chain. This temperature inside feeders was confirmed prior to experiments by highly accurate temperature probe (Radboudumc instrumental service, The Netherlands). Mosquitoes were allowed to feed for up to 15 min. Unfed mosquitoes were removed from the cup with an aspirator and placed in another cup where they were killed by 70% ethanol; fully fed mosquitoes were supplied with 10% glucose solution and held in the temperature and humidity controlled room.

### Mosquito preparation for dissection

At 9–10 days post-infection, mosquitoes were anesthetized by freezing at − 20 °C for 5 min. They were then immersed in 70% ethanol for 5 s and transferred to a Petri dish filled with RPMI medium [27] before dissection. The mosquito thorax was held with ultra-fine tweezers while an insulin needle or scalpel was used to make two incisions at the second and last abdominal segment. The abdomen epithelium was gently pulled off the mosquito abdomen in a single motion. The midgut thereby remained attached to the immobilized thorax. Malpighian tubules and ovaries were removed and put aside as they might interfere with the oocysts reading if they overlap the midgut. Dissections were performed in 0.5% mercurochrome under a stereo-microscope; staining was allowed for approximately 10 min and then the gut was transferred into a drop of RPMI and covered by a cover slip. The advantage of staining is that oocysts absorb the dye differentially from the midgut tissue, with oocysts appearing darker and allowing characteristic features of malaria parasites to be more readily recognized. Dissected mosquito midguts were examined for presence of oocysts, using light microscope at 10× and confirmed at 40× magnification.

### Supportive experiments using in vitro cultured parasites

To support the experiments conducted in Nagongera, membrane–feeding experiments were also conducted at Radboud University Medical Center to obtain high-resolution images of oocysts and oocyst-like structures. For this, *P*. *falciparum* parasites (NF54 line) were cultured as described elsewhere [28] and used in mosquito feeding assays with *Anopheles stephensi* (Sind-Kasur Nijmegen strain) [29]. On day 10, mosquitoes were dissected and different concentration of mercurochrome (0.1%, 0.5%, 1%) were used to stain the midguts. Midguts were moved to a drop of RPMI before examination by microscopy and taking high-resolution images (Axio Cam MRc-5, Zeiss, Germany).

## Results

### Challenges in mosquito husbandry

**Fig. 1:**
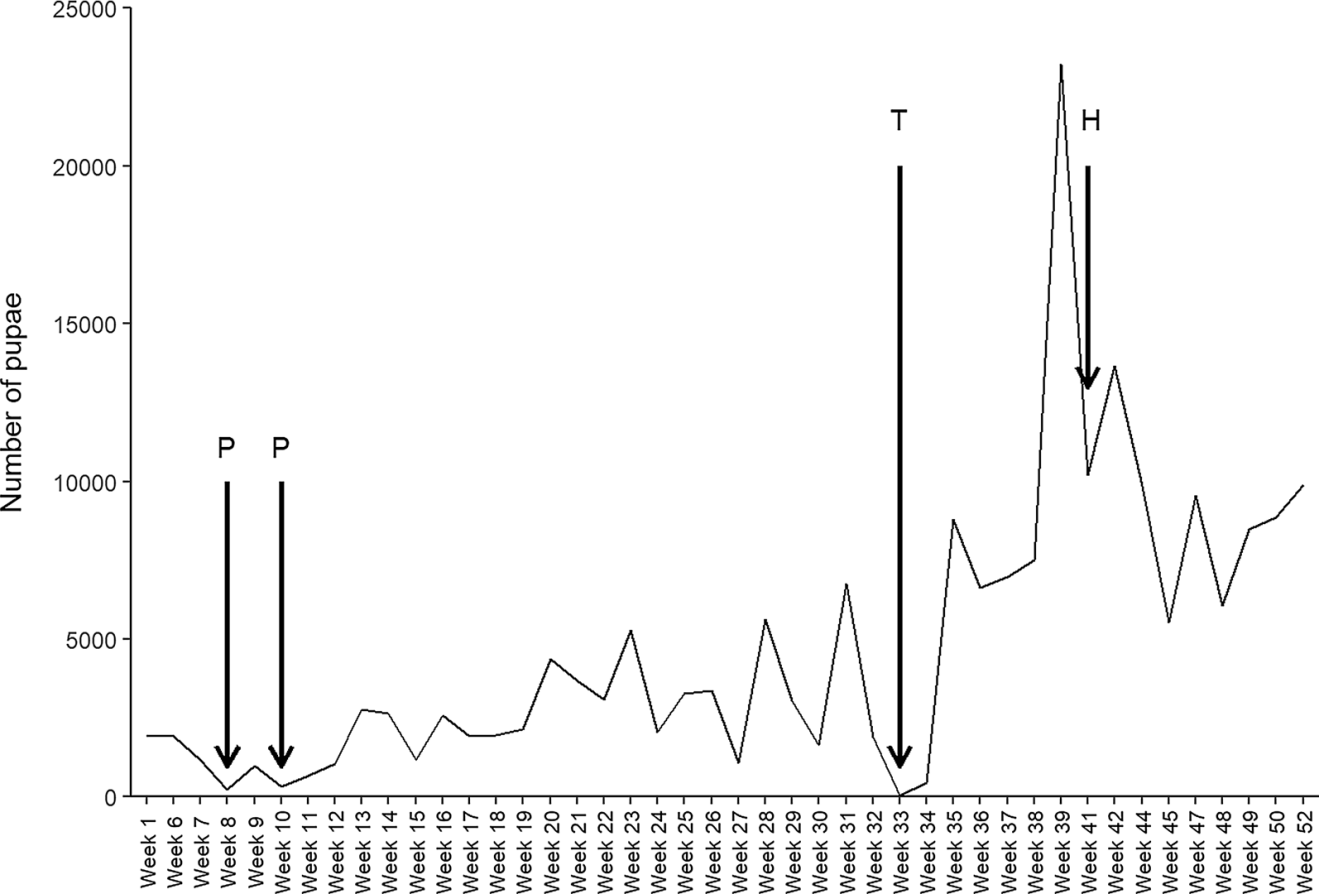
Weekly pupae production in the Nagongera insectary during the year 2018. Total weekly number of pupae is indicated on the Y-axis against time on the X-axis. Letters indicate occurrences that significantly affected pupae production. P-Drop in pupae production due to pathogens (microsporidia), T-Drop in pupae production due to high temperature in adult room that killed almost all adults hence allowed no egg production, H-Intentionally reduced colony production to respond to limited cage-space for adult mosquitoes

**Fig. 2:**
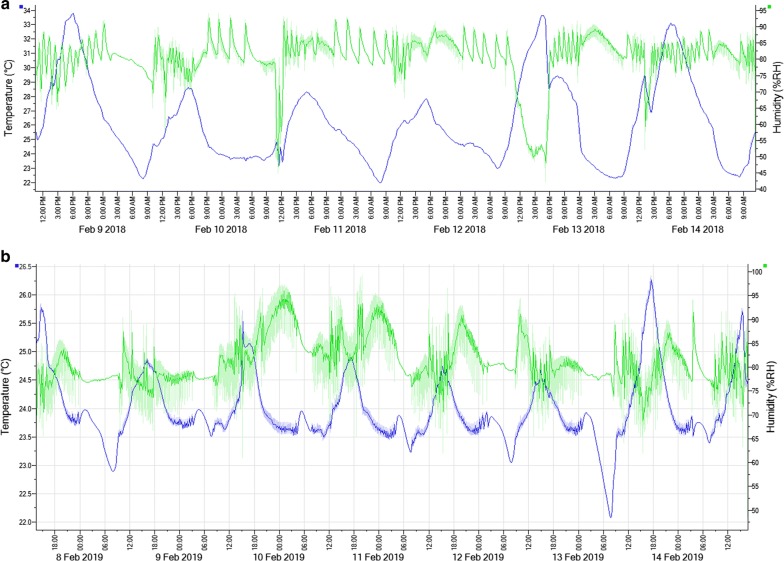
Illustrative fluctuations in temperature and humidity in the adult room of the insectary. Temperature and humidity logs from a TinyTag probe showing a period of unstable temperature and % relative humidity in the insectary (**a**) and showing a period with relatively stable temperature and % relative humidity in the insectary (**b**)

The insectary was established in 2014 and mosquitoes were reared to support DMFA from 2016. The production ranged between 5000 and 10,000 adult mosquitoes per week, with marked variation and several moments when production dropped (Fig. 1). One of the factors contributing to these fluctuations was maintenance of temperature within the targeted range of 25–27 °C. During the rainy seasons (March–May and August–October), outside temperature regularly fell to ~ 17–20 °C at night, resulting in large fluctuations in temperature inside the insectary (Fig. 2a). Locally purchased heaters controlled by thermostats were used periodically, to improve temperature stability. From 6 p.m. to 6 a.m., heaters were switched on for 20 min per hour; the same approach was used for larvae rooms. Whilst this improved temperature control (Fig. 2b), on one occasion the thermostat failed to switch off the heater automatically and caused temperature to rise beyond the maximum set temperature (up to 40 °C), causing mortality in almost the entire colony (Fig. 1).

Infestation of ants (Formicidae) was another major challenge for the colony. To prevent ants from reaching the colony, water and oil were placed in buckets beneath racks with mosquito cages. In addition, sugar pads were placed in trays on the floor of the adult mosquito holding room to lure invading ants to an alternative source of nutrients. Sugar-attracted ants were killed under running water and sugar pads were replaced on a daily basis to avoid fungi formation. On two occasions, bees also invaded the adjacent insectary used for resistance studies. The beehive was removed but during one instance insecticides were sprayed to remove the bees completely, also affecting the insectary used for the current experiments. This resulted in considerable mortality and a drop in colony numbers.

### Pathogen infection larval and adult stages

**Fig. 3:**
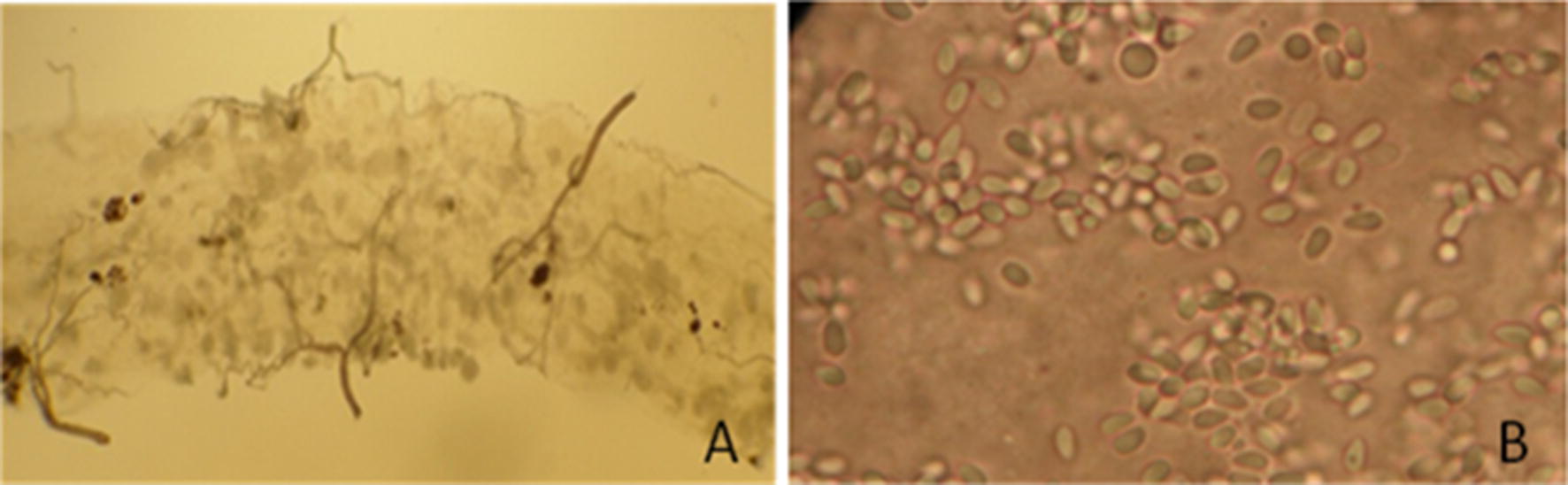
Images of midgut*s* of dissected mosquitoes infected with microsporidia spores. Depicted are images taken during microscopy at ×10 magnification of a mosquito midgut infected with microsporidia spores (**A**) or ×40 magnification of microsporidia spores (**B**)

Infection of the colony by other pathogens was another challenge. Microsporidia may exert a pathogenic effect in 4th instar larvae, manifested by increased mortality. Affected larvae have a lumpy white appearance due to the presence of spores in the thorax and abdomen. In mild infections, larvae can still pupate and mature into adults. However, midguts show a “grayish” appearance at 10× while at 40× spores are more evidently present (Fig. 3). Fungi zoospores may also increase larvae mortality and appear as ovoid pointed motile biflagellate structures swimming in proximity of larvae. Therefore, wet preparations were prepared by adding a small drop of water on a slide and by placing dead larvae or a dissected mosquito midgut covered with a cover slip and examining tissue for evidence of infection. If cages with adult mosquitoes infected either with microsporidia or fungi were identified, they were discarded. Mild infection of larval stages was accepted but monitored closely. Hygiene measures were imposed by changing water in the larvae basins once every 3 days, keeping larvae food in the fridge, washing basins with soap (Marseilles—72% olive oil, pH neutral, no additives and naturally biodegradable) and spraying basins in between use with 70% ethanol to prevent pathogen growth.

### Classification of oocysts and artifacts

**Fig. 4:**
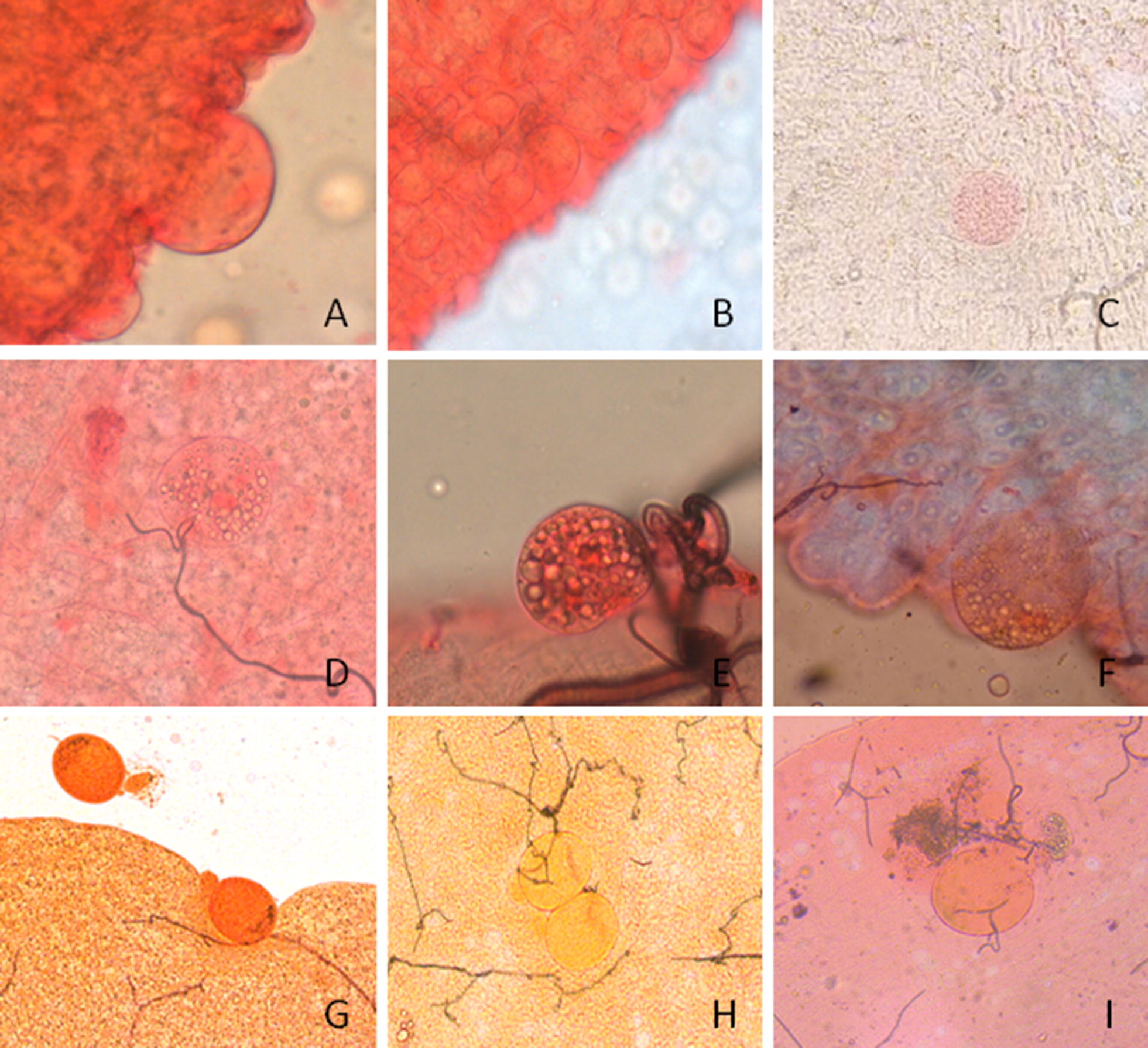
Oocyst-like structures stained with different concentrations of mercurochrome at different magnifications. Protruding epithelial cell ×40, stained with 0.5% mercurochrome (**A**). Epithelial cells nuclei ×20, these cells have no black pigment and no defined membrane (**B**). Air bubble stained in 0.1% mercurochrome, at ×10 (**C**). Detached midgut cell at ×40, stained with 0.1% mercurochrome (**D**, **E**). Immature detached mosquito eggs on midgut. At ×40 magnification and 0.1% mercurochrome (**F**), at ×20 magnification and 1% mercurochrome (**G**), at ×20 magnification and 1% mercurochrome (**H**), and at ×40 magnification and 1% mercurochrome (**I**)

**Fig. 5:**
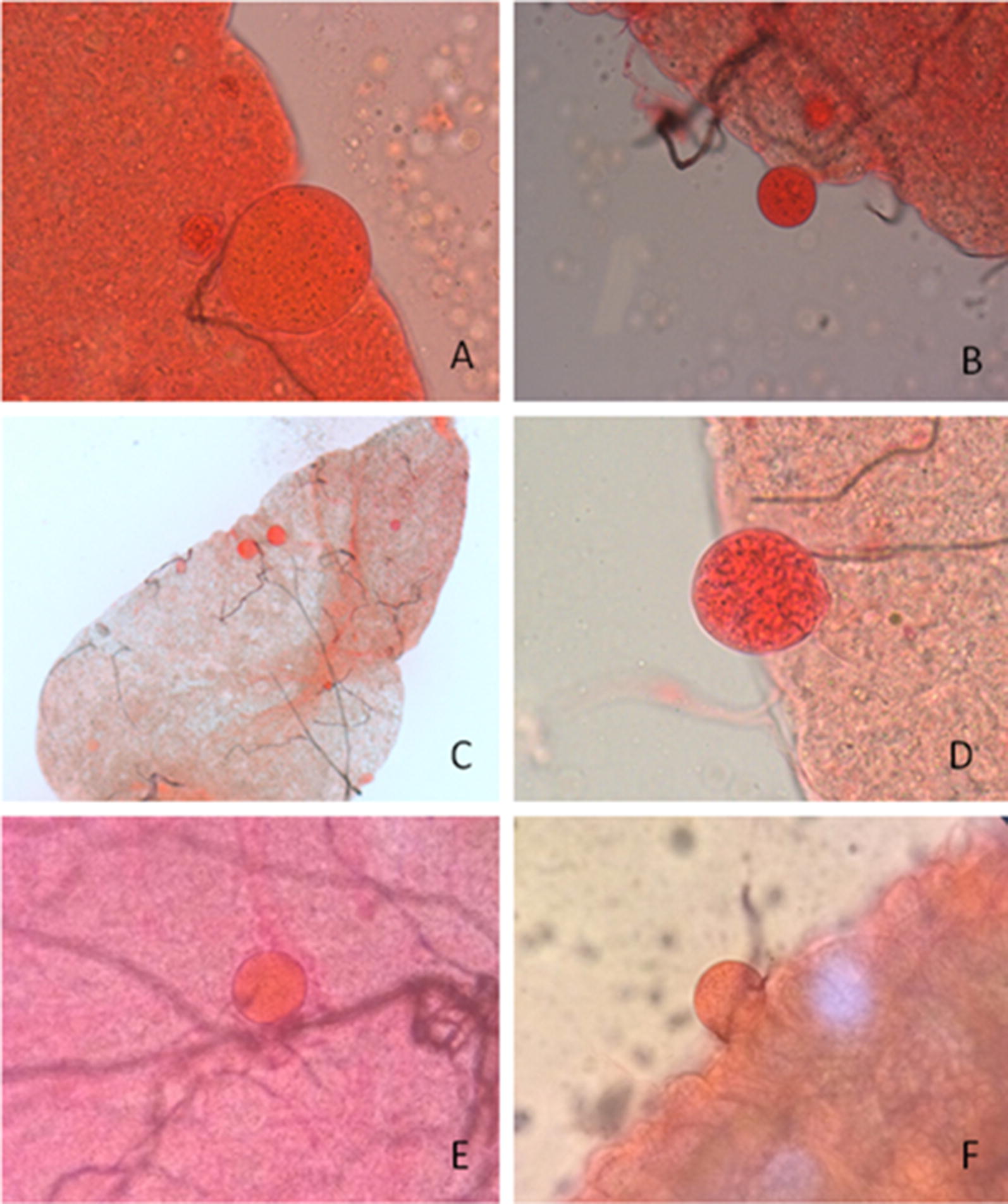
Features of oocysts stained with different concentrations of mercurochrome at different magnifications. Oocyst at ×40 magnification and 0.5% mercurochrome (stained for 20 min) with clear presence of pigment (**A**). Oocyst at ×20 magnification and 0.5% mercurochrome (stained for 10 min) (**B**). Oocyst at ×5 magnification and 0.1% mercurochrome (stained for 15 min) (**C**). Oocyst at ×40 magnification and, 0.1 mercurochrome (stained for 15 min) (**D**). Oocysts at ×10 magnification and, 0.5% mercurochrome. Pictures taken in the field with a smart phone camera (**E**, **F**)

Oocysts were classified using the following diagnostic features: (i) have to be spherical structures within the mosquito midgut or on the peripheral tissue, (ii) need to have a clearly defined round edge, (iii) are always attached to the epithelium, rarely seen on the foregut and (iv) have pigment granules in the cytoplasm. These criteria allowed differentiation between oocysts and other structures that resemble oocysts such as epithelial cell nuclei, protruding epithelial cells, air bubbles, immature floating eggs or invagination of the epithelial wall (Fig. 4). Clean dissection was considered particularly important to ensure that no other parts of mosquitoes were included on a slide for oocyst screening. The presence of Malpighian tubules or pieces of ovaries sometimes resulted in detached undeveloped eggs that are spherical with a defined edge, often of the same size of a mature oocyst (Fig. 4F–H). Sometimes dark pigment was observed in these eggs, typically filling only half of the egg (Fig. 4G). Rolling the midgut by moving the cover slip with a needle until the presumed oocyst protruded from the external surface of the midgut was useful in these instances to better visualize the pigment and rule out the presence of air bubbles or other artifacts. Artifacts can be spherical or ovoid in shape and of variable size, sometimes the same size as mature oocysts. Several features of common artifacts are described in Table 1. The main distinguishing feature between artifacts and mature oocysts was the presence of defined pigment within the oocysts. Staining concentrations resulted in different appearances of guts and oocysts (Fig. 5). In our protocol, 0.5% mercurochrome and 10-min staining were considered ideal to stain (but not over-stain) guts and clearly visualize oocysts and parasite pigment.

**Table 1:** Common artifacts observed in mosquitoes midguts that are often mistaken for oocysts

Artifacts	Picture	Features
Epithelial cells	a–b	No black pigment, not differential staining
Air bubble	c	No black pigment Does not absorb the staining well
Invagination of midgut wall	d–e–f	No black pigment, bubbles inside, asymmetry
Detached ovary egg	g–h–i	Recognized by the presence of a little knob; pigment covers only one side of the egg; they absorb the staining differentially from the midgut tissue

Reference to pictures in Fig. 4

## Discussion

Membrane feeding experiments are important for understanding human-to-mosquito transmission and the human infectious reservoir for malaria. However, identifying oocysts in dissected mosquitoes is challenging especially if transmission intensity is low and therefore infection rates are low. Here, experiences and challenges were presented that were faced when establishing an insectary and conducting DMFAs in Nagongera, a rural sub-county in Uganda where malaria control has intensified. The aim of this methods paper was to provide a supportive document for other researchers working in similar settings and provide examples of correct dissection and staining procedures and strict adherence to diagnostic criteria to allow robust outcome assessments.

The presented work identified the maintenance of temperature, pest infestations and pathogen infections as important challenges to generate a healthy mosquito colony for experiments. Even when a healthy colony is achieved and maintained, a number of factors determine the optimum time to dissect mosquitoes following membrane feeding. Ambient temperature is one of the most important factors that influence the sporogonic cycle. Since mosquitoes are poikilothermic and thus incapable of maintaining the temperature of their body constant, fluctuations in ambient temperature may affect both parasite development and mosquito survival. This implies that any variation in temperature during day or night can accelerate, slow down or even completely prevent sporogonic development. Whilst the permissive temperature for *P. falciparum* development ranges from 16 to 32 °C [30], lower temperatures increase the time needed to complete development [31–33]. High temperatures may be detrimental for early sporogonic development. Mosquitoes exposed to a temperature of 30 °C in the period between ingestion of gametocytes, zygote formation and migration of ookinetes tend to have lower oocyst development with complete developmental arrest in some instances [33]. It is, therefore, important to maintain relatively constant temperature post-infection experiments and in particular to avoid high temperatures shortly after feeding. The current experiences also demonstrated the risk of crashing mosquito colonies due to extremely high temperatures in the adult holding room (40 °C in the current study). By installing a thermostat, the insectary temperature was maintained at a range that was deemed appropriate for the current purposes although fluctuations remained inevitable. Oocyst maturation starts 2 days after gametocyte infection and is completed around 10–14 days under ideal laboratory conditions at 26 °C and 80% relative humidity. On day 2–3, oocysts have an average diameter of 7 µm, this size reaches more than 40 µm by day 9–10 when they are mature and ready to release sporozoites [34]. Oocyst development time differs between *Plasmodium* species with *Plasmodium vivax* reaching maturation in 9 days, *Plasmodium falciparum* in 11–12 days, *Plasmodium ovale* 14–15 days, followed by the slow growing parasite *Plasmodium malariae* that takes up to 21 days to complete maturation [35, 36]. The ideal time point for dissection thus has to strike a balance between size (later time points allow easier identification), mosquito survivorship (decreasing at later moments) and occupational safety (dissection has to occur prior to sporozoite release unless additional safety precautions are in place). Experienced microscopists in laboratories with highly controlled conditions typically dissect *P. falciparum* infected midguts 6–7 days post infection to assess the presence of oocysts. In the current study, dissecting around day 9–10 was considered useful as earlier dissections gave smaller oocysts that were more difficult to detect. In general, larger oocyst size may better allow detection of sporadic oocysts as occurring after feeding on blood containing low gametocyte densities [14, 37, 38]. In the current experiments in Nagongera, dissecting mosquitoes 9–10 days after incubation at ~ 25–27 °C and 80% relative humidity was considered optimal.

In addition to temperature fluctuations, investing pests have to be avoided at all costs. Although this sounds evident, it is often challenging in rural conditions. Moreover, mosquito pathogens pose a considerable challenge. Entomopathogenic fungi, and protozoa can affect mosquito survivorship in aquatics stages, reduce the lifespan and fecundity of adult mosquitoes and, perhaps most challenging, affect susceptibility to *Plasmodium* infection and interfere with midgut examination [39–45]. During experiments in Nagongera, microsporidia and fungi zoospores were occasionally detected in larvae. However, these infections were not to a level likely to affect transmission experiments, as was plausibly the case in the heavily infected tissues observed elsewhere (Fig. 3) [43]. In these infections, microsporidia may not only reduce mosquito susceptibility but pansporoblasts on the midgut surface may also be confused for oocysts due to their round shape. They can be differentiated from oocysts as they contain spores that are rice-like shaped and not translucent like malaria oocysts.

Other ovoid structures resembling oocysts are very common in mosquito midguts [39], but some have peculiar morphological details that help to differentiate them from oocysts. However, some artifacts can mislead even the most experienced microscopist. For such misleading artifacts, confirmation by nested PCR [46] or preferably qPCR [47, 48] should be conducted. Alternative methods for the detection of parasites in mosquitoes have been proposed recently, including CSP-ELISA [47], CSP-slotblot [49, 50], nested PCR [46] and qPCR [47–49]. None of these approaches allow reliable quantification of oocysts at present or a direct assessment of infectivity in the field. Of note, protein-based detection methods (CSP-ELISA and slotblot) are incompatible with mercurochrome staining whilst PCR is possible after initial staining and microscopy examination [49]. To allow examination of oocyst density, microscopy remains of great value, provided it is carefully performed by experienced microscopists able to identify oocysts with all their characteristics. In case of doubt, and during early phases of studies when microscopists are acquiring skills and experience, confirmation of a subset of infected guts by alternative methods is important. Otherwise, misclassification is likely to occur where individuals may be incorrectly identified as infectious to mosquitoes. This would lead to an overestimation of the human infectious reservoir for malaria.

## Conclusion

Microscopy remains the most widely used method to identify and quantify *Plasmodium* infection in mosquitoes. In this study, other oocyst-like structures were described in detail in order to support other researchers in how to recognize artifacts. Extensive training of insectary staff allows meaningful assessments of human to mosquito transmission to support assessments of the human infectious reservoir for malaria.

## Data Availability

Data are available upon reasonable request by an email to the corresponding author.

## Abbreviations

ACT: artemisinin-based combination therapy
CSP: circumsporozoite protein
DMFA: direct membrane feeding assays
ELISA: enzyme-linked immunosorbent assay
IPTp: intermittent preventive therapy in pregnancy
IRS: indoor residual spraying
LLIN: long-lasting insecticidal nets
PCR: polymerase chain reaction
*Pf*aEIR: *Plasmodium falciparum* annual entomological inoculation rate
qPCRv: quantitative polymerase chain reaction
RDTs: rapid diagnostic tests
